# Effect of the Combination of Vanillin and Chitosan Coating on the Microbial Diversity and Shelf-Life of Refrigerated Turbot (*Scophthalmus maximus*) Filets

**DOI:** 10.3389/fmicb.2020.00462

**Published:** 2020-03-31

**Authors:** Tingting Li, Xiaojia Sun, Haitao Chen, Binbin He, Yongchao Mei, Dangfeng Wang, Jianrong Li

**Affiliations:** ^1^Key Laboratory of Biotechnology and Bioresources Utilization, Dalian Minzu University, Ministry of Education, Dalian, China; ^2^College of Food Science and Technology, Bohai University, Jinzhou, China; ^3^Food Safety Key Lab of Liaoning Province, Jinzhou, China; ^4^National and Local Joint Engineering Research Center of Storage, Processing, and Safety Control Technology for Fresh Agricultural and Aquatic Products, Jinzhou, China; ^5^Beijing Key Laboratory of Flavor Chemistry, Beijing Technology and Business University, Beijing, China

**Keywords:** high-throughput sequencing, quality, shelf-life, turbot filets, vanillin–chitosan coating

## Abstract

The effect of the combination of vanillin and chitosan (VC) coating on the microbiota composition and shelf-life of turbot (*Scophthalmus maximus*) filets during a 15-day storage period at 4 ± 1°day was investigated in this study. The control and coated fish samples were analyzed periodically for sensory and chemical attributes [total volatile basic nitrogen (TVB-N), thiobarbituric acid reactive substances (TBARS), and pH] and the presence of dominant spoilage microbiota. The results suggested that the sensory and the chemical quality of turbot filets effectively improved after treatment with vanillin (final concentration 2 mg/ml) combined with 1% chitosan, and the shelf-life was prolonged for 6 to 7 days compared with the control group. Furthermore, high-throughput sequencing showed that *Proteobacteria* (52.2%) and *Firmicutes* (29.8%) were the dominant bacteria at the phylum level in fresh turbot filets, while *Pseudomonadaceae* (40.2%) and *Lactobacillaceae* (39.4%) were the dominant bacteria at the family level in deteriorated turbot filets. However, after VC treatment, the relative abundance of *Pseudomonadaceae* and *Lactobacillaceae* decreased significantly due to the growth inhibition of potential bacteria, specifically spoilage bacteria, along with the rich bacterial diversity at the end of storage. Therefore, our data indicated that VC treatment might be effective in decreasing bacteria-induced quality deterioration and in extending the shelf-life of refrigerated turbot filets.

## Introduction

Turbot (*Scophthalmus maximus*) is a highly nutritious, economically important fish species. However, the sensory properties of fish deteriorate with storage, processing, and distribution, owing to chemical and biological changes (Özogul et al., 2006). Globally, the annual damage rate of aquatic product harvests is estimated to be 10% (Kulawik et al., 2013). Among many elements, microorganisms are considered to play a key role in the fish spoilage process (Remenant et al., 2015). From the perspective of sensory rejection, the most active microorganisms producing enough metabolites were considered as specific spoilage organisms (SSOs), which involved more than one microbial group, genus, or species in most cases (Don et al., 2018). Thus, controlling the existence and storage conditions of microorganisms is an important factor for determining the fresh fish quality and shelf-life.

In recent years, many studies have shown that most chemical antioxidants may be carcinogenic and toxic at high concentrations (Barbosa-Pereira et al., 2013). Hence, bio-based coating preservation, as an eco-friendly and alternative way to improve aquatic product quality, continues to attract attention (Dehghani et al., 2017; Costa et al., 2018). Recently, chitosan has been a research hotspot, owing to its good biodegradability and film forming properties. Being the only alkaline natural product, chitosan is the second largest natural polymer after cellulose and is widely used in food preservation (Shariatinia and Jalali, 2018). Vanillin (4-hydroxy-3-methoxybenzaldehyde) is a key component of natural vanilla extracts applied in foods, beverages, and pharmaceuticals (Fache et al., 2016).

Previous studies have confirmed that vanillin, as an antimicrobial and an antioxidant, enhances the antibacterial effect in chitosan membranes and inhibits spoilage bacterial growth (Cava-Roda et al., 2012; Zou et al., 2015). Due to its special aromatic structure, vanillin has been successfully incorporated into other new polymers to expand its application (Li et al., 2016; Kamaraj et al., 2018). Furthermore, Stroescu et al. (2015) indicated that chitosan–vanillin composites had good antimicrobial properties. To the best of our knowledge, no study has focused on the quality changes and microbial diversity of vanillin–coated chitosan on refrigerated turbot filets using high-throughput sequencing.

To date, high-throughput sequencing is most commonly employed in microbial ecology, which focused on providing a cost-effective means for identifying complex microbial communities (Reuter et al., 2015). This method can deeply analyze bacterial community and diversity and has been widely used in many studies such as aquaculture environment and sewage and soil treatment (Fierer et al., 2012; Shu et al., 2015; Zheng et al., 2017). In this study, the effect of vanillin–chitosan (VC) treatment on sensory scores, pH, total volatile basic nitrogen (TVB-N), and thiobarbituric acid reactive substances (TBARS) on turbot filets stored at 4 ± 1°C was studied. Furthermore, the dynamic changes in the microbial communities and dominant microbiota in turbot filets were characterized by high-throughput sequencing combined with microbial enumeration.

## Materials and Methods

### Fish Sample Preparation

Live cultured turbots (average lengths and weights were 34.00 ± 2.03 cm and 920.00 ± 22.37 g, respectively) were purchased from the local aquatic market in Jinzhou, Liaoning Province, China, in October and slaughtered (stunned on the head, bled, and gutted) by professional personnel. The fish, covered with crushed ice, were transferred to the laboratory at Bohai University within 20 min, and the turbots were fileted by hand and washed with water.

### Edible Coating Solution Preparation

Vanillin (purity >96%) and chitosan powder extracted from crabs were purchased from Senfu Natural Products Co. (Shangluo, Shanxi, China). Chitosan solution (1%, w/v) was added to 1% acetic acid (v/v) and stirred at 28°C for 2 h, and then vanillin at 2.0 mg/ml was homogenized with the chitosan solution. The final solution was stirred for 2 h to achieve complete dispersion and immediately used for coating, according to the method of Li et al. (2012) with a slight modification.

### Treatment of Fish Samples

The dimensions of each filet were approximately 5 cm × 2 cm × 1.5 cm, and they were randomly divided into two groups: one was immersed in VC solution for 5 min, and the other was immersed in sterile water for 5 min (control group). The samples were then dried at 4°C in a sterile incubator for 90 min, as previously reported (Zhang et al., 2015). Finally, all the samples were individually packed in aseptic retort pouches and stored for up to 18 days at 4°C before use. Biochemical testing was carried out in triplicate on days 0, 3, 7, 9, 12, and 15. However, high-throughput sequencing was only done on storage days 0, 7, and 15.

### Sensory Evaluation

Sensory analysis was performed according to the method described by Fan et al. (2008). Seven trained panelists from the laboratory staff (three females and four males between 25 and 40 years old) evaluated filet color, odor, flavor, general acceptability, and texture using a nine-point hedonic scale (1–9; 1 being extremely disliked and 9 being extremely liked). A sensory score of 4 was the acceptable borderline.

### Chemical Analyses

Briefly, 5 g of minced filet was homogenized in 45 ml of distilled water. After letting it stand at room temperature for 30 min, the pH of each sample was measured using a pH meter (pHFE20, Mettler Toledo) following the Chinese standard (GB/T5009. 24-2003).

The minced filet (10 g) was transferred to the distilling tube of a Kjeltec 8400 (FOSS, Hiller, Denmark), followed by 1 g of MgO and 50 ml of distilled water. TVB-N (mg N/100 g muscle) values were measured using the Kjeltec 8400 (FOSS, Hiller, Denmark) in Kjeldahl mode, as previously reported (Lv et al., 2018).

Thiobarbituric acid (TBA) value was determined based on a colorimetric method, with slight modification (Xiong et al., 2015). The minced filet (10 g) was homogenized with 25 ml of distilled water and 25 ml of 10% trichloroacetic acid. The resulting mixture was centrifuged at 3,000 *g* for 10 min. The supernatant (5 ml) was then added into 5 ml of 0.02 M TBA solution and incubated in a boiling water bath for 40 min. Equivalent quantities of sterile water and of the TBA solution were used as control. Absorbance at 532 nm was measured using a Shimadzu UV-2550 spectrophotometer (Jiangsu, China). A standard curve for calculating malonaldehyde (MDA) concentration was obtained using 1,1,3,3-tetrameth-oxypropane, and the TBA value was expressed in mg MDA/kg sample.

### Microbiological Analysis

Ten grams of minced filet was aseptically transferred to an aseptic retort pouch containing 90 ml of sterile NaCl (0.85%) and 0.1% peptone water and homogenized with a stomacher for 60 s. A 10-fold dilution series was prepared in sterile physiological saline solution, and 0.1 ml of each dilution was spread-plated in triplicate (PCA, Aoboxing, China), following the method described by Li et al. (2017). Total viable counts (TVC) were measured using the pour plate method, and incubation was at 28°C for 48 h. Meanwhile, *Pseudomonas* spp. were incubated on cephaloridine fucidin–cetrimide selective agar at 28°C for 48 h, *Aeromonas* were incubated on the *Aeromonas* medium base agar at 28°C for 48 h, *Enterobacteriaceae* were incubated on violet red glucose agar at 28°C for 48 h, H_2_S-producing bacteria were incubated on iron agar at 28°C for 72 h by counting black colonies only, and lactic acid bacteria were incubated on Man Rogosa Sharpe agar at 28°C for 72 h. The results represented the mean log CFU g^–1^ ± standard deviation of four replicates. All agar used in this study were purchased from Haibo Biotechnology Company (Qingdao City, China).

### DNA Extraction and High-Throughput Sequencing

#### DNA Extraction and PCR Amplification

Genomic DNA was extracted according to the method described by Armougom and Raoult (2009). DNA purity and concentration were verified by agarose gel electrophoresis. The DNA sample was diluted with sterile water to 1 ng/μl. The prepared samples were transported to Nuohezhiyuan (Dalian, China) for further experiment. The universal primers 357 F (5′-CCTACGGGAGGCAGCAG-3′) and 926 R (5′-CCGTCA ATTCM TTTRAGT-3′) were employed to target the V3–V4 region of 16S rRNA gene. The PCR solution (25 μl) consisted of 2.5 μl 10 × PCR buffer, 1 μl of template DNA, 1 μl each of the forward and the reverse primers, 2.5 mM of dNTP mixture, 1.5 U (5 U/μl) of Taq DNA polymerase, and 17.2 μl of ddH_2_O. The reaction conditions were as follows: 5 min at 95°C, followed by 25 cycles of incubation at 95°C for 30 s, annealing at 55°C for 30 s, and extension at 72°C for 40 s, and followed by a final extension at 72°C for 8 min, using the Phusion^®^ High-Fidelity PCR Master Mix (New England Biolabs Inc., Ipswich, MA, United States). The PCR products were assessed using 2% agarose gel, and the qualified products (bright main strip between 400 and 450 bp) were purified with the QIAquick Gel Extraction Kit (Qiagen, Valencia, CA, United States). Finally, the DNA library was constructed using the TruSeq^®^ DNA PCR-Free Sample Preparation Kit. The constructed library was quantified by Qubit and Q-PCR and sequenced using HiSeq2500 PE250.

#### High-Throughput Sequencing Data Analysis

The paired-end reads were joined using FLASH (V1.2.7^1^) and the low-quality sequences removed. According to the QIIME’s Tags quality control^2^, the raw tags need to be quality-filtered to obtain high-quality clean tags. The clean tags were matched with the database and the chimera sequences were then removed using the UCHIME algorithm^3^. All effective tags were clustered into operational taxonomic units (OTUs) at 97% sequence similarity. The taxonomic assignment was carried out using the Ribosomal Database Project classifier with a confidence threshold of 1.0. Various calculations were performed, including the community richness (Chao1 and ACE), the depth of sequencing (Good’s coverage), and the diversity of the bacterial community (Shannon index).

### Statistical Analysis

All experiments were conducted in triplicate. The data collected were analyzed using variance analysis (ANOVA), followed by Tukey’s multiple comparison test. SPSS 14.0 procedures (SPSS Inc., Chicago, IL, United States) were used to determine statistically significant differences.

## Results and Discussion

### Sensory Evaluation

**FIGURE 1:**
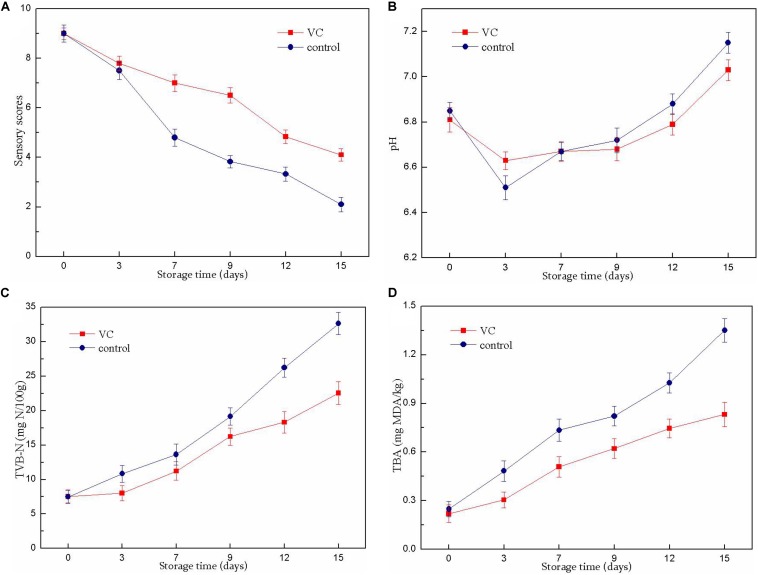
Changes in the sensory scores **(A)**, pH value **(B)**, TVB-N value **(C)**, and TBA value **(D)** of turbot filets with or without vanillin–chitosan (VC) treatment during refrigeration (4°C). VC represents 2.0 mg/ml vanillin and 1% (w/v) chitosan coating treatment.

The results of the sensory evaluation of turbot filets are shown in Figure 1A. The fish samples were considered to be acceptable for consumption until the sensory score reached 4 (Fan et al., 2008). The sensory scores of all samples showed a decreasing trend during refrigerated storage (15 days). The unacceptable scores (3.83) in the control group were obtained on day 9 of storage, while the sensory scores of VC treatment were consistently >4 during the entire storage period. Compared with the control group, VC treatment significantly (*P* < 0.05) prolonged the shelf-life of turbot filets for 6 to 7 days. Sensory deterioration was mainly caused by microbial spoilage and oxidation reactions. Thus, these results were consistent with the results of chemical and microbiological analysis. These results demonstrated that the decrease in sensory quality loss might be related to the inhibition of microbial growth and lipid oxidation by VC coating, thereby prolonging the shelf-life of turbot filets during refrigeration. This observation was similar to that of Yu et al. (2018b), who observed that the sensory quality of grass carp filets treated with chitosan composite coatings was improved during refrigerated storage and had significantly extended shelf-life compared with the control group.

### pH Analysis

The changes in the pH values of the turbot filets during refrigeration are shown in Figure 1B. The pH values of all samples decreased slightly at the beginning of storage and gradually increased in the later stages. The decrease in the pH values in the control samples might be attributable to the release of inorganic phosphate and the accumulation of lactic acid at the beginning of storage, while the slight decrease in VC samples might be related to the coatings (pH 6.65 ± 0.04) formed on the surface of the filets. However, the later increase was probably caused by the accumulation of alkaline compounds with the extension of storage time. Nevertheless, there was no significant difference between the two samples.

### TVB-N Analysis

Total volatile basic nitrogen was used as an important indicator to assay fish freshness; it rapidly accumulated in post-mortem fish muscle under refrigerated conditions due to protein degradation by the spoilage bacteria. The changes of TVB-N values in the turbot filet during refrigeration are displayed in Figure 1C. In this study, the initial TVB-N value was 7.52 (control) and 7.48 (VC) mg N per 100 g of filet. However, a sharp rise in the TVB-N value was noticed in all samples after the 7th day. A TVB-N value of 25 mg N/100 g indicated unsuitability for human consumption (Ojagh et al., 2010). This level was exceeded on the 12th day in the control group, while the value of the VC group remained below this level throughout the storage period, indicating that the VC treatment effectively delayed filet quality deterioration during refrigeration. The higher viable count value of the uncoated sample could explain the higher TVB-N value due to the TVB-N value of fish that was closely related to the decomposition process of microorganisms. In addition, these results suggested that the decrease in the TVB-N value of filets with VC treatment might involve the increased antimicrobial activity of chitosan combined with vanillin; the reduction in the ability of bacteria to oxidative deamination was caused by chitosan as well. Similarly, Qiu et al. (2014) reported that the TVB-N values significantly decreased after chitosan and citric acid or licorice extract treatment, and the growth of microorganisms was also inhibited correspondingly, indicating that the combinations could significantly extend the shelf-life of Japanese sea bass fish filets during refrigerated storage.

### TBARS Analysis

The TBA value is an index to measure the degree of lipid oxidation, evaluated by the MDA content, which is a secondary product of lipid oxidation. As shown in Figure 1D, the TBA value of the fresh filets was low but increased with storage time. The increase in the TBARS value might involve the partial dehydration of the fish and increased unsaturated fatty acid oxidation (Nowzari et al., 2013). The TBA value increased more rapidly in the control group than that of the VC group; the TBA value of the control group was 1.351 mg of MDA/kg and the value was 0.832 mg of MDA/kg for the VC group on day 15. Therefore, VC treatment had a significant (*P* < 0.05) inhibitory effect on lipid oxidation and delayed the quality deterioration of turbot filet. Recently, chitosan coatings and films were considered to be good barriers to oxygen permeation, and the chitosan coating applied directly to the surface of the fish might act as a barrier between the fish and its surrounding air, thereby slowing the diffusion of oxygen to the surface of the fish (Sathivel et al., 2007; Li et al., 2013). In addition, vanillin was also considered to have good oxidation resistance. Therefore, these results suggested that the reduction in TBA value might be related to the enhanced antioxidant activity of chitosan combined with vanillin, thus inhibiting lipid oxidation in turbot filets. This result was consistent with the report of Souza et al. (2010) who found that the TBA values in chitosan-coated salmon filets were lower than those of the uncoated filets throughout storage, suggesting that the coating treatment effectively prevented lipid oxidation.

### Microbial Analysis

Listed in Table 1 are the results of the microbial communities found, including *Pseudomonas* spp., *Aeromonas* spp., *Enterobacteriaceae*, H_2_S-producing bacteria, lactic acid bacteria, and TVC. The initial count of *Pseudomonas* spp. was 3.15 log CFU/g; a similar result was reported by Boziaris et al. (2013). The initial counts of *Aeromonas* spp. and H_2_S-producing bacteria such as black colonies, possibly *Shewanella putrefaciens*, were 2.97 and 3.46 log CFU/g, respectively. By the 7th day of storage, the counts of all microbial communities and TVC remained <6 log10 CFU/g, implying a slow rate of increase. With the prolongation of storage time, the counts of *Pseudomonas* spp., H_2_S-producing bacteria, and lactic acid bacteria increased sharply. Meanwhile, the lowest count was detected in *Pseudomonas* spp. The counts of VC treatment were 6.49 log CFU/g at the end of storage, indicating the significant (*P* < 0.05) inhibition of bacterial growth. Other studies had reported that vanillin could inhibit the growth of spoilage bacteria by affecting the integrity of the cytoplasmic membrane, resulting in loss of ion gradients (Fitzgerald et al., 2010). Moreover, the counts of *Pseudomonas* spp., H_2_S-producing bacteria, and lactic acid bacteria were 8.69, 8.51, and 8.49 log CFU/g, respectively, and became the potential SSOs in turbot filets. However, it was well known that the culture media is not possibly selective enough. Thus, 16S amplicon sequencing was used to study the microbial diversity, composition, and potential SSO in turbot filets.

**TABLE 1:** Changes in the microbial communities of turbot filets with VC and control during refrigeration.

**Bacteria**	**Sample**	**0**	**3**	**9**	**12**	**15**
*Pseudomonas*	Control	3.15 ± 0.11a	3.88 ± 0.11b	6.08 ± 0.13d	7.45 ± 0.19e	8.69 ± 0.21g
	VC	3.02 ± 0.21a	3.79 ± 0.16ab	5.47 ± 0.16c	6.18 ± 0.09d	6.49 ± 0.11e
*Aeromonas*	Control	2.97 ± 0.11a	3.41 ± 0.16b	5.77 ± 0.31e	7.02 ± 0.16e	8.06 ± 0.27f
	VC	3.08 ± 0.30a	3.43 ± 0.21ab	5.09 ± 0.14c	6.41 ± 0.08d	7.41 ± 0.94e
*Enterobacteriaceae*	Control	3.15 ± 0.29a	4.09 ± 0.26ac	6.11 ± 0.16e	7.66 ± 0.16f	8.09 ± 0.19g
	VC	3.11 ± 0.22ab	3.97 ± 0.22b	5.21 ± 0.24bc	5.97 ± 0.21c	7.02 ± 1.22d
H_2_S-producing bacteria	Control	3.46 ± 0.19b	4.15 ± 0.31bc	5.99 ± 0.22e	7.31 ± 0.25f	8.51 ± 1.97g
	VC	3.29 ± 0.16a	3.75 ± 0.19ab	5.31 ± 0.28c	6.28 ± 0.16d	7.05 ± 0.27f
Lactic acid bacteria	Control	3.47 ± 0.31b	3.94 ± 0.29ac	6.09 ± 0.19e	7.59 ± 0.09f	8.49 ± 0.18g
	VC	3.09 ± 0.25a	3.74 ± 0.27bc	4.91 ± 0.24d	5.64 ± 0.16e	7.04 ± 0.09ef
TVC	Control	3.22 ± 0.16ab	3.84 ± 0.22b	5.80 ± 0.26d	7.18 ± 0.08f	8.74 ± 0.16g
	VC	3.26 ± 0.24ab	3.88 ± 0.16b	5.37 ± 0.27bc	6.18 ± 0.27e	8.51 ± 0.13f

*All values are expressed as the mean ± standard deviation of three values. Different letters in the same microbial enumeration between control and VC are significantly different (*P* < 0.05).*

### Sequencing and Bacterial Composition Analysis

#### Analysis of Illumina MiSeq Data

The bacterial diversity of turbot filets with or without VC treatment during refrigeration was described through 16S rRNA sequencing. Illumina Hiseq sequencing generated a total of 66,351, 133,905, and 130,232 raw sequence reads from fresh, control, and VC samples, respectively. Considerable values (309,690) of effectiveness were obtained after double-ended read splicing. The effective tags were then clustered into OTUs. Table 2 shows the alpha diversity indices of each sample, including the OTU number, Simpson index, Chao1 index, and Good’s coverage. The values of Good’s coverage were ≥0.998 in all samples, which indicated that the information was sufficient to reveal the microbial phylotypes in turbot filets. Additionally, the OTUs were lower than the ACE and Chao1 index, indicating that most bacterial diversity was obtained by our analysis. Shannon index reflects the diversity of the bacterial community, and the highest Shannon indices were observed in VC compared to that of the control on day 15, indicating that the microbial communities in filets after VC treatment had higher bacterial diversity.

**TABLE 2:** Alpha diversity estimation in turbot filets with (VC) or without (control) vanillin-chitosan treatment.

**Sample**	**Raw reads**	**Clean reads**	**OTU**	**Shannon**	**ACE**	**Chao1**	**Simpson**	**Good’s coverage**
Fresh	66351	61299	587	0.919	603.652	655.309	0.919	0.999
Control 7d	62372	59809	487	0.725	547.772	537.808	0.725	0.999
Control 15	71533	67156	231	0.695	299.074	291.551	0.809	0.998
VC7d	58271	54269	377	0.908	479.052	484.158	0.696	0.999
VC15d	71962	67157	357	0.808	382.289	377.153	0.808	0.999

**FIGURE 2:**
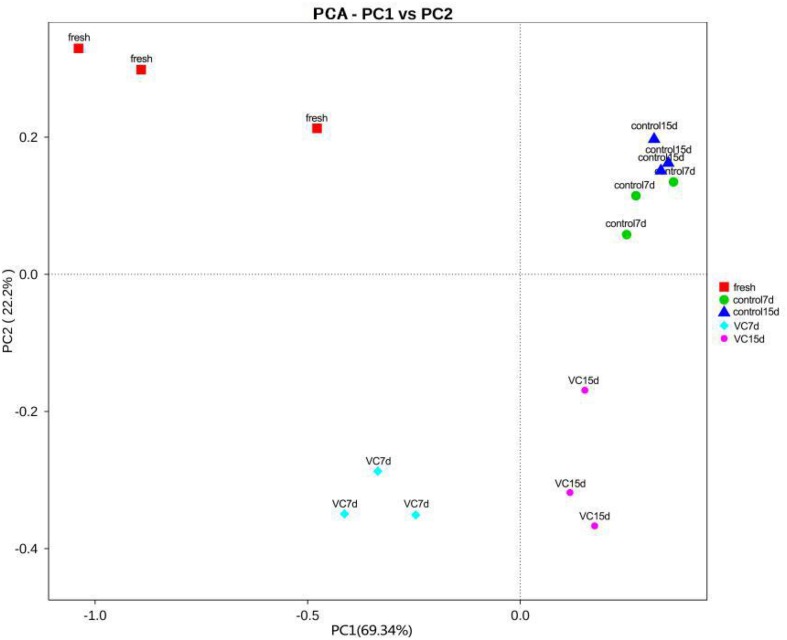
Results of principal component analysis at the genus level for the treatment and the control groups in turbot filets on weighted unifraction distance during refrigeration.

Principal component analysis was commonly performed to classify and cluster samples (Parlapani et al., 2018). The main changes in the bacterial diversity of the different samples accounted for 91.54% (PC1 69.34% and PC2 22.2%) at the genus level (Figure 2), implying a good separation by region. An obvious region discrepancy between the VC and the control groups revealed the different microbial communities, which might be related to the antibacterial activity of the VC coating. Moreover, the dots corresponding to control on days 7 and 15 were located in a cluster, suggesting the microbial composition.

#### Bacterial Diversity and Richness

**FIGURE 3:**
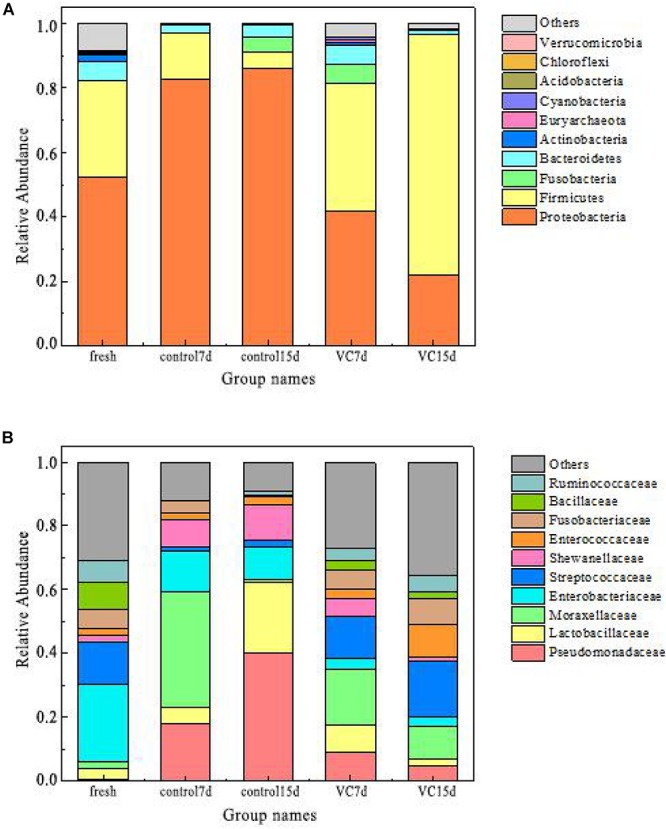
Relative abundance at the phylum level **(A)** and at the family level **(B)** in turbot filet samples. VC represents vanillin–chitosan treatment with turbot filets during refrigeration.

The relative abundance values of the dominant microbiota (top 10) at the phylum and the family levels are presented in Figures 3A,B. The predominant phyla in fresh turbot filets belonged to *Proteobacteria* (52.2%) and *Firmicutes* (29.8%) at the phylum level. Also, Zhang et al. (2017) reported that the most dominant phyla in vacuum-packaged common carp after cinnamon essential oil treatment were *Proteobacteria* and *Firmicutes*. For the control samples, with the extension of storage time, the proportion of *Proteobacteria* increased from 52.2 to 86.2% and it was the predominant bacterial phyla, while the proportion of *Firmicutes* decreased from 29.8 to 4.8%. With regard to VC treatment, a decrease was observed in the proportion of *Proteobacteria*; however, a dramatic increase was observed in the proportion of *Firmicutes*, accounting for 74.6% at the end of storage.

The abundance of the dominant bacterial family is shown in Figure 3B. In the fresh samples, *Enterobacteriaceae* (24.2%) and *Streptococcaceae* (13.5%) were predominant populations, followed by *Bacillaceae*, *Ruminococcaceae*, and *Fusobacteriaceae*. With increased storage time, *Moraxellaceae* (36.2%) were the dominant microbial species in the stored control samples at day 7. However, *Pseudomonadaceae* (40.2%) and *Lactobacillaceae* (39.4%) became the predominant bacterial family in the control samples on day 15, followed by *Shewanellaceae* (10.9%). In the VC samples, a significant decrease was observed in the proportion of *Pseudomonadaceae*, accounting for 4.8% on day 15. Many studies have determined that *Pseudomonadaceae* and *Shewanellaceae* were the most dominant family in fish samples (Nuin et al., 2008; Yu et al., 2018a). Additionally, some studies have reported that *Lactobacillus* may be related to the metabolism of amino acid metabolism, owing to the ability to produce several antimicrobial metabolites (Quijada et al., 2017). In this study, *Pseudomonadaceae* and *Lactobacillaceae* might be the main spoilage microorganisms, and VC coating effectively inhibited their family levels.

**FIGURE 4:**
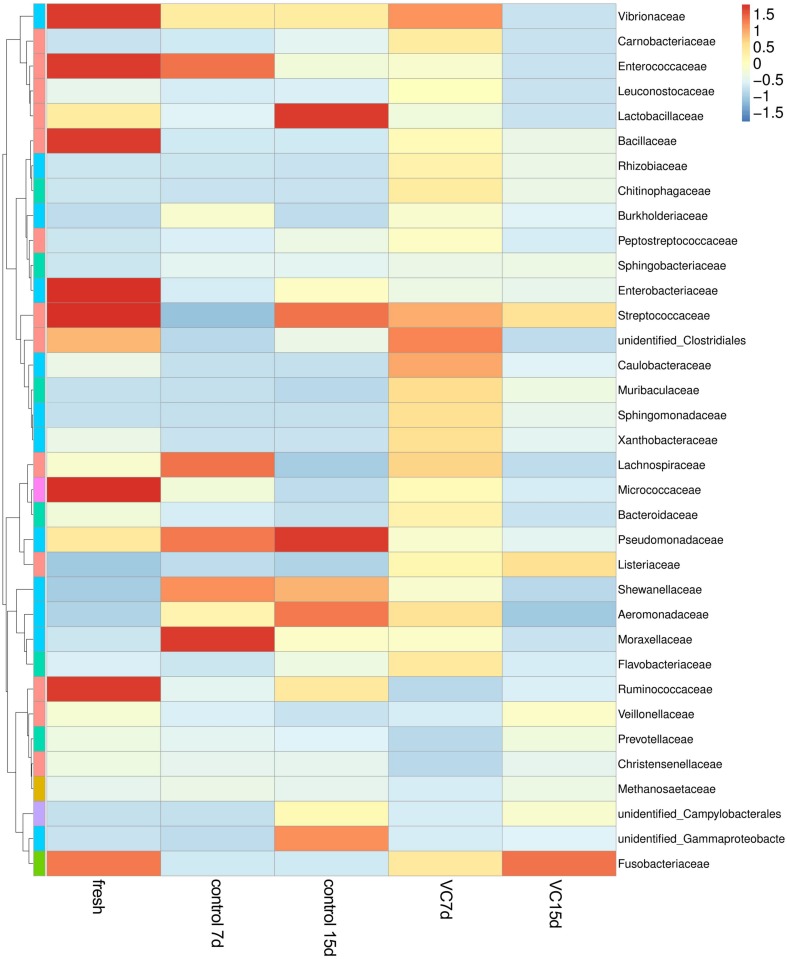
Heat map of microorganism species abundance at the family level of turbot filets during refrigerated storage. The color intensity is proportional to the relative abundance in each row, and VC represents 2.0 mg/ml vanillin and 1% chitosan coating treatment of turbot filets.

A heat-map expressing the relative abundance of the dominant genera (top 35) was constructed to analyze the dynamics and the composition of the microbial communities in the different samples (Figure 4). Among the top 35 family-level phylotypes, red represented the higher relative abundance. The most diverse bacterial composition was observed in fresh turbot filets and mainly included *Vibrionaceae*, *Enterococcaceae*, *Bacillaceae*, *Enterobacteriaceae*, *Streptococcaceae*, and *Ruminococcaceae*. However, the most dominant populations were *Lactobacillaceae* and *Pseudomonadaceae* and the secondary dominant populations were *Streptococcaceae*, *Shewanellaceae*, and *Aeromonadaceae* in the control samples at the end of storage. Additionally, more diverse microbial communities were observed in turbot filets treated with VC. The relative abundance of *Pseudomonadaceae* and *Lactobacillaceae* in the VC group was low at the end of storage, and a significant (*P* < 0.05) difference in the microbial community structure was observed between the control and the VC groups.

## Conclusion

An in-depth understanding of the microbial diversity in turbot filets contributes to the application of appropriate strategies for quality improvement and shelf-life extension for these highly perishable products. This study mainly investigated the effect of VC coating on the microbial diversity and shelf-life of turbot filets during refrigerated storage. High-throughput sequencing analysis indicated that the main spoilage microorganisms in turbot filets were *Lactobacillaceae* and *Pseudomonadaceae*, both in lower relative abundance after VC treatment. In addition, chemical and sensory analysis demonstrated that VC treatment inhibited lipid oxidation, protein decomposition, and microbial growth and enhanced the sensory quality of turbot filets. These findings suggested that VC coating might effectively reduce the quality deterioration induced by bacteria and prolong the shelf-life of turbot filets for 6 to 7 days during refrigeration. A further study needs to understand the potential mechanism of the effect of VC coating on spoilage microorganisms and to apply this technology to the fish preservation industry.

## Data Availability Statement

All datasets generated for this study are included in the article/supplementary material.

## Author Contributions

TL and XS contributed to the conception of the study. XS performed the data analyses and wrote the manuscript. HC, BH, YM, and DW contributed significantly to the analysis and manuscript preparation. JL helped to perform the analysis with constructive discussions. All authors contributed to the manuscript revision, and read and approved the submitted version.

## Conflict of Interest

The authors declare that the research was conducted in the absence of any commercial or financial relationships that could be construed as a potential conflict of interest.

## Footnotes

1 http://ccb.jhu.edu/software/FLASH/

2 http://qiime.org/scripts/split_libraries_fastq.html

3 http://www.drive5.com/usearch/manual/uchime_algo.html
